# 1st Global Consensus for Clinical Guidelines for the Rehabilitation of the Edentulous Maxilla: Patient and Cross‐Disciplinary Expert Single‐Round Surveys

**DOI:** 10.1111/clr.70023

**Published:** 2026-02-24

**Authors:** Guo‐Hao Lin, Giulia Brunello, Ronald E. Jung, Ina Kopp, Frank Schwarz, Hom‐Lay Wang, Franz J. Strauss

**Affiliations:** ^1^ Department of Orofacial Sciences University of California San Francisco School of Dentistry San Francisco California USA; ^2^ Department of Periodontics & Oral Medicine University of Michigan School of Dentistry Ann Arbor Michigan USA; ^3^ Department of Oral Surgery University Hospital of Düsseldorf Düsseldorf Germany; ^4^ Charité‐Universitätsmedizin Berlin, Corporate Member of Freie Universität Berlin and Humboldt‐Universität zu Berlin Department of Orthodontics and Dentofacial Orthopaedics Berlin Germany; ^5^ Clinic of Reconstructive Dentistry, Center of Dental Medicine University of Zurich Zurich Switzerland; ^6^ AWMF‐Institut für Medizinisches Wissensmanagement Philipps‐Universität Marburg Marburg Germany; ^7^ Department of Oral Surgery, Implantology and Oral Medicine Goethe University Frankfurt am Main Germany; ^8^ Center for Studies and Innovation in Dentistry, Faculty of Dentistry Universidad Finis Terrae Santiago Chile

**Keywords:** dental implants, patient participation, stakeholder participation, survey

## Abstract

**Objectives:**

To gather perspectives from patients and cross‐disciplinary experts on treatment practices and outcomes in managing the edentulous maxilla to inform a structured consensus process and highlight areas for future research.

**Materials and Methods:**

An 18‐item single‐round survey was distributed in November 2024 with 68 patients and 68 cross‐disciplinary experts. Participation was voluntary, anonymous, incentive‐free, and required informed consent. Consensus was defined as > 75% and ≤ 95% agreement or disagreement, and strong consensus as > 95% agreement or disagreement.

**Results:**

Response rates were 60.3% among patients, and 30.9% among cross‐disciplinary experts. Consensus or strong consensus was achieved by patients on 15 statements across nine items and by cross‐disciplinary experts on 15 statements across eight items. Items reaching consensus included the use of cone‐beam computed tomography, preference for fixed full‐arch prostheses, major treatment outcomes, need for hard tissue augmentation, home care regimens, dental implant lifespan, procedure difficulty, cost‐effectiveness, and patient‐reported outcomes. Both groups also highlighted the need for further research on full‐arch implant rehabilitation of the maxilla.

**Conclusion:**

Findings from both surveys inform the development of clinical practice guidelines in treating the edentulous maxilla by providing valuable perspectives from patients and cross‐disciplinary experts. Areas lacking consensus illustrate real‐world variations in treatment approaches, highlight knowledge gaps in the current literature, and reveal important differences among patient expectations, expert opinions, and available evidence.

## 1 Introduction

Edentulism remains a major global health concern, particularly among older adults, many of whom require prosthetic rehabilitation (Collaborators et al. 2020; Curtis et al. 2021). While most studies on edentulous patients focus on clinician‐reported outcomes (ClinROs) (U.S. Food and Drug Administration 2021) such as implant and prosthesis survival (Messias et al. 2023; Messias et al. 2021), these ClinROs often overlook the patient's perspective (Chalmers and Glasziou 2009).

In recent years, increasing attention has been directed toward patient‐reported outcomes (PROs) (Calvert et al. 2018; Johnston et al. 2019). These are subjective evaluations of health status and treatment effectiveness, typically collected using standardized questionnaires known as patient‐reported outcome measures (PROMs) (Weinfurt and Reeve 2022). PROMs are designed to reflect the patient's lived experience, offering valuable insights into both the challenges and perceived benefits of implant therapy (Sanz et al. 2023; Tonetti, Sanz, et al. 2023).

The divergence between the outcomes valued by patients and those prioritized by clinicians (Thoma and Strauss 2022) highlights the importance of involving a broad range of stakeholders in determining which outcomes to measure and why. Historically, consensus and position statements related to the treatment of the edentulous maxilla have been classified as S1 level (“The 6th EAO Consensus Conference, 10–12 February 2021, Virtual Meeting,” Clinical Oral Implants Research 2021; Hämmerle et al. 2018; Wismeijer and Chen 2018; Wismeijer et al. 2023). This level of consensus involves a selected group of experts without a formal methodology or the involvement of key stakeholder groups (AWMF 2023). According to the United States Food and Drug Administration (FDA), stakeholders include a wide range of individuals and groups with an interest in healthcare decisions, including but not limited to, clinicians, patients, and industry representatives (U.S. Food and Drug Administration 2021).

Incorporating diverse stakeholders input is essential to improving care for patients with an edentulous maxilla. It supports the creation of inclusive, structured consensus guidelines, ensuring that practical considerations are addressed from multiple viewpoints and that all relevant evidence is thoroughly evaluated (Williamson et al. 2017). A diverse and balanced consensus group also reduces the potential for bias stemming from individual or organizational interests. In fact, the broader the representation within the group, the more likely it is that the resulting consensus will be widely accepted and implemented (Williamson et al. 2017; Williamson et al. 2012).

To address the clinical challenges associated with edentulous maxillae, the first Global Consensus for Clinical Guidelines (GCCG) was established under the initiative titled “Patient‐Centered Clinical Workflow in Implant Dentistry”. This initiative brought together leading international experts and multi‐stakeholder participants to develop a structured consensus addressing key clinical questions (Graham et al. 2011).

The aim of this report was to gather insights from patients and cross‐disciplinary experts, including clinical research directors/professionals of leading companies, organizations, and foundations in the field of implant dentistry and regenerative biomaterials, regarding treatment practices and outcomes in the management of the edentulous maxilla. These insights will guide a structured consensus process and help identify areas where additional research is needed.

## 2 Materials and Methods

The current patient and cross‐disciplinary expert surveys employed a single‐round method for effective group‐based judgment and decision‐making in healthcare research (Boulkedid et al. 2011; Hasson et al. 2000). This technique aims to gather diverse perspectives while minimizing drawbacks of group discussions, such as dominance by certain individuals or opinions (Barrett and Heale 2020).

An expert panel was assembled to ensure broad multi‐stakeholder representation comprising three groups:

1. Clinicians: Selected to ensure wide‐ranging expertise and geographic representation in implant dentistry.
2. Patients: Individuals with lived experience of receiving dental implants to rehabilitate an edentulous maxilla.
3. Cross‐disciplinary experts: Clinical research directors and professionals from leading companies and foundations specializing in implant dentistry and regenerative biomaterials.

This study reports on the findings from the patient and cross‐disciplinary expert surveys. The protocol for this study was approved by the Ethics Committee of the University of Düsseldorf (Protocol no. 2024‐2973_1). The study was conducted and reported in accordance with the criteria outlined in “Good practice in the conduct and reporting of survey research” (Kelley et al. 2003).

### 2.1 Study Design

Patient and cross‐disciplinary expert surveys were carried out as a single‐round survey in preparation for the GCCG Workshop, scheduled for June 2025 in Boston, USA. The study aimed to explore emerging trends and advancements in the rehabilitation of the edentulous maxilla while addressing gaps between common clinical practices and scientific evidence. By gathering input from patients and cross‐disciplinary experts, the study aimed to outline current implant treatment strategies, identify gaps in the literature, and highlight discrepancies between stakeholder perspectives and existing evidence.

The findings from these single‐round surveys were used to inform the development of key questions for further investigation through systematic reviews or structured consensus during the GCCG Workshop. The final survey questionnaires were developed and approved by the Scientific Chairs of the GCCG (Frank Schwarz, Hom‐Lay Wang), in collaboration with the Cross‐disciplinary Expert and Patient Panel (Franz‐Josef Strauss, Guo‐Hao Lin) and the Survey Panel (Giulia Brunello, Todd Schoenbaum).

The GCCG Scientific Task Force engaged expert clinicians to recruit edentulous individuals who had been rehabilitated with full‐arch implant‐supported prostheses for participation in the patient survey. Each clinician was asked to select one eligible patient from their practice to complete the survey. Cross‐disciplinary experts, such as clinical research directors and professionals from leading companies specializing in implant dentistry and regenerative biomaterials, were selected based on their expertise and invited directly by the Scientific Task Force. Only one individual from each company was invited to prevent over‐representation of specific viewpoints.

### 2.2 Questionnaires

The patient and cross‐disciplinary expert questionnaires were designed based on the latest literature, focusing on the rehabilitation of the edentulous maxilla using dental implants. The initial draft was formulated by the Cross‐disciplinary Expert and Patient Panel.

To ensure clarity and relevance, the questionnaires underwent an iterative validation process, involving multiple rounds of feedback and revisions. In addition to the Scientific Chairs, contributors included the Cross‐disciplinary Expert and Patient Panel and the Survey Panel. Their input helped refine the questionnaire before obtaining ethical approval.

The patient and cross‐disciplinary expert questionnaires were created in English and designed to be completed in approximately 15–20 min. For the patient survey, the lay language without dental jargon was implemented for comprehensive understanding of the survey content. The patient questionnaire was translated into several languages for distribution to ensure the comprehensive understanding of the questions by the patient participants. The final version, consisting of 18 items for each questionnaire (Appendix S1 for patient questionnaire, Appendix S2 for cross‐disciplinary expert questionnaire), was organized as follows:

Consent

- Declaration of consent (item 1);

Treatment planning

b Use of cone‐beam computed tomography (CBCT) scans (items 4–5);
c Preference for removable vs. fixed full‐arch prosthesis (item 2);
d Major treatment outcomes (item 3);
e Distal extension considerations for full‐arch restorations (item 6);
f Preference for provisional prosthesis (item 8);

Treatment procedures

g Hard tissue augmentation (item 7);
h Preferred impression techniques (item 13);

Maintenance care

i Frequency for regular check‐ups (item 9);
j Home care and occlusal guards (items 10–11);
k Potential complications (item 14);
l Patient communication (item 15);
m Life expectancy for dental implants (item 12);

Fundamental outcomes to be included in future studies

n Future research on maxillary full‐arch rehabilitation with dental implants (item 16);
o Relevant patient‐reported outcomes (PROs) (item 17);
p Difficulty of procedures and treatment cost‐effectiveness (item 18).

### 2.3 Sample Size, Expert Selection, and Data Collection

**FIGURE 1:**
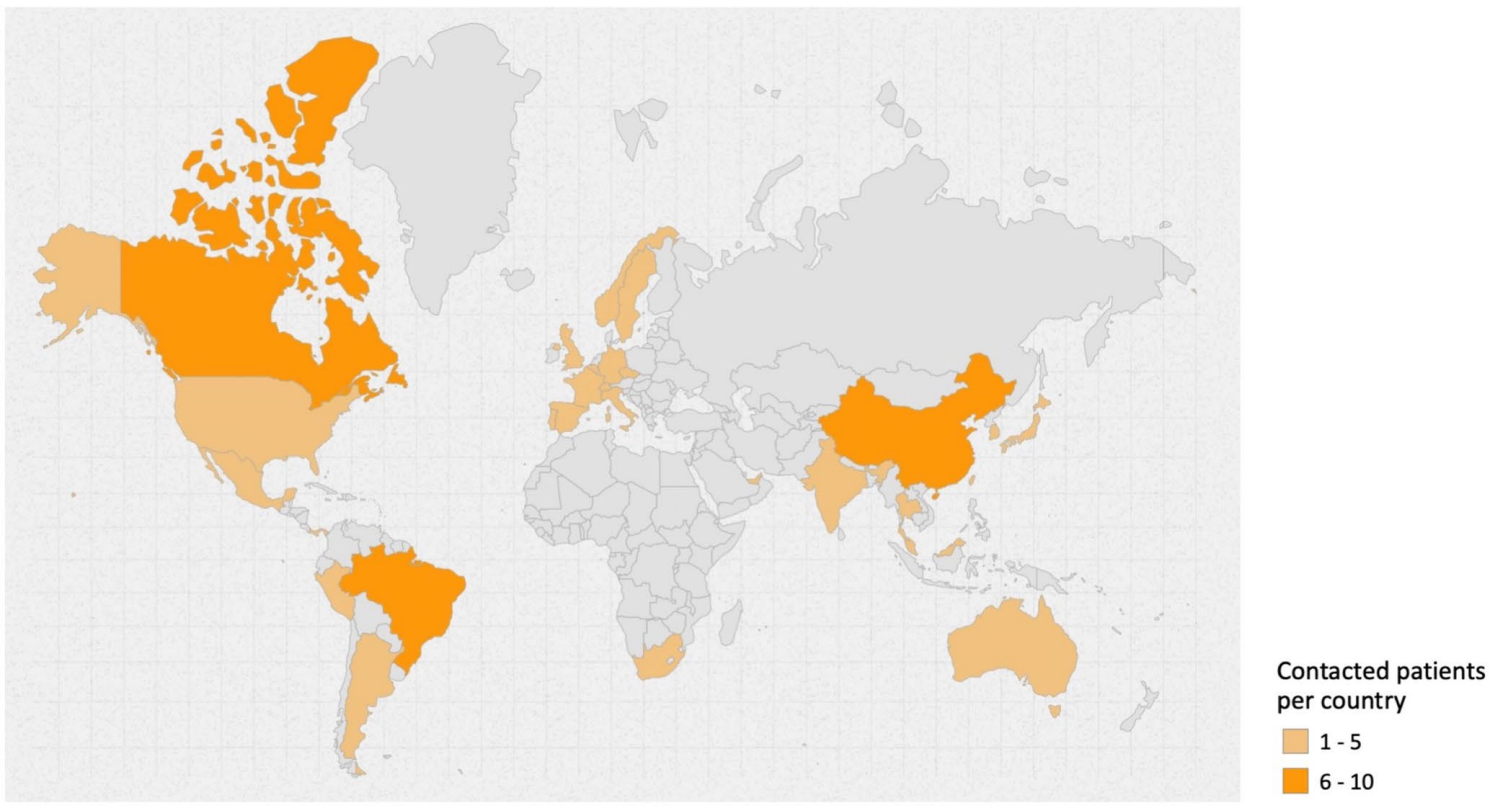
Distribution by country of the contacted patients. Map created using the open source online tool available at https://www.amcharts.com/ (amCharts, Neringa, Lithuania).

**FIGURE 2:**
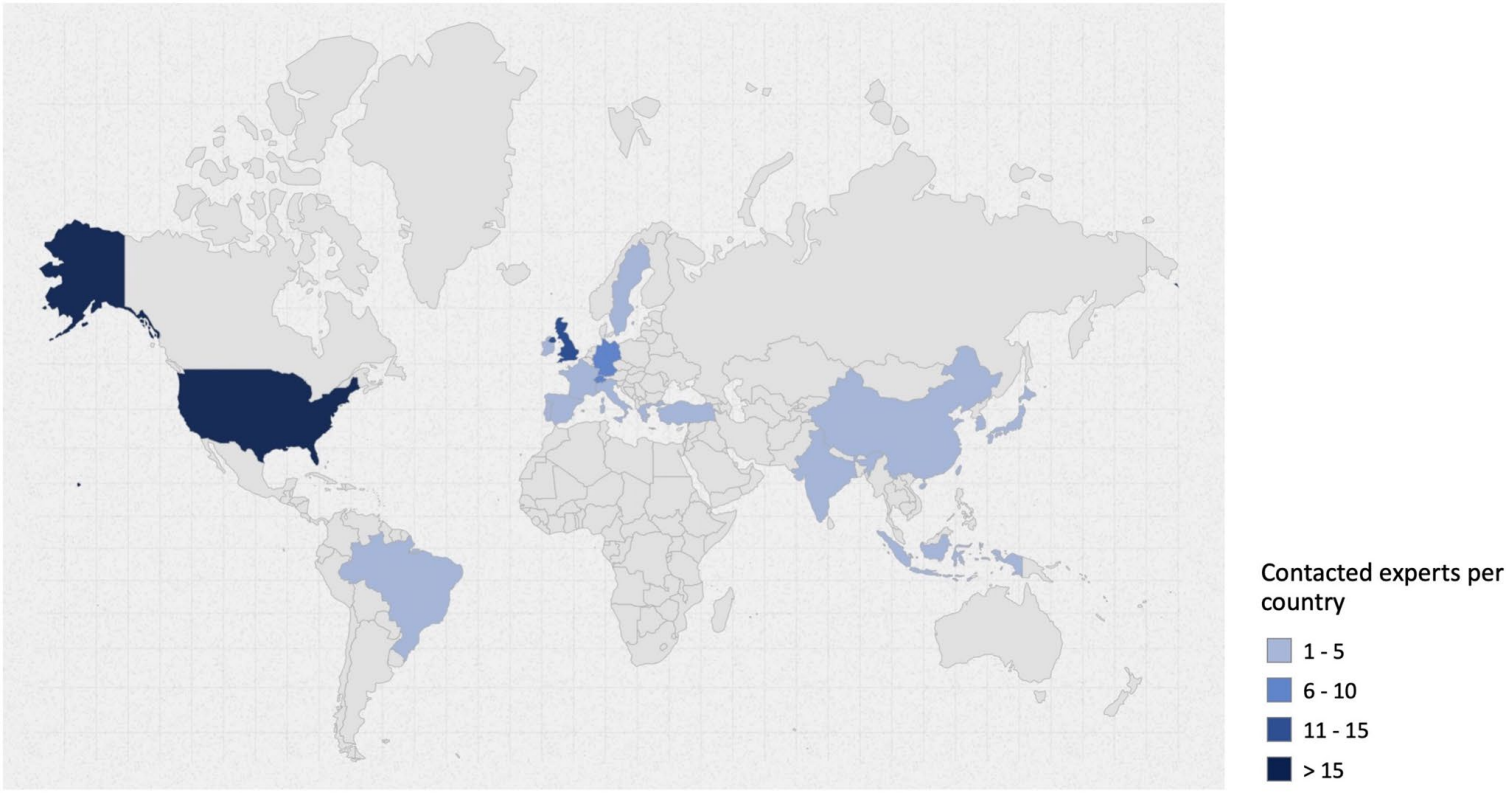
Distribution by country of the contacted cross‐disciplinary experts. Map created using the open source online tool available at https://www.amcharts.com/ (amCharts, Neringa, Lithuania).

There is no universally established formula for calculating the sample size in survey studies; however, expectations are often guided by the COMET Initiative guidelines (Williamson et al. 2017). For survey studies limited to individual stakeholder groups, such as patients and cross‐disciplinary experts, a sample size of 30 per group may be sufficient (Manyara et al. 2024). Therefore, 68 patients and 68 cross‐disciplinary experts were contacted. A detailed breakdown of the patients by country is provided in Table 1, Figure 1. A breakdown of these stakeholders by country is provided in Table 2, Figure 2. Because the data was collected anonymously, it was not possible to distinguish between respondents and non‐respondents. The European Association for Osseointegration (EAO) Office distributed the survey links via email to the responsible clinicians, who then invited their patients and directly to the cross‐disciplinary experts selected and approved by the Scientific Task Force. To avoid over‐representation of specific viewpoints, each clinician was limited to identifying a single patient to participate in the survey. All included patients had edentulous maxillae and had received either implant‐supported or non‐implant‐supported full‐arch maxillary prostheses. They demonstrated a clear understanding of the available treatment options, including the concepts, benefits, and limitations of each. All patients completed the surveys independently without any influence from the clinicians.

**TABLE 1:** Details and distribution by country of the contacted patients.

Country	No.	%
Argentina	3	4.4
Australia	2	2.9
Belgium	1	1.5
Brazil	8	11.8
Canada	6	8.8
China	8	11.8
Czech Republic	1	1.5
France	1	1.5
Germany	1	1.5
India	4	5.9
Italy	1	1.5
Japan	2	2.9
Korea (Republic of)	1	1.5
Malaysia	2	2.9
Mexico	3	4.4
Norway	1	1.5
Panama	1	1.5
Peru	1	1.5
Portugal	2	2.9
Singapore	3	4.4
South Africa	1	1.5
Spain	1	1.5
Sweden	1	1.5
Switzerland	2	2.9
Taiwan	3	4.4
Thailand	1	1.5
United Arab Emirates	1	1.5
United Kingdom	1	1.5
United States of America	5	7.4

**TABLE 2:** Details and distribution by country of the contacted cross‐disciplinary experts.

Country	No.	%
Brazil	1	1.5
China	1	1.5
France	1	1.5
Germany	6	8.8
Greece	1	1.5
India	4	5.9
Indonesia	1	1.5
Ireland	1	1.5
Italy	2	2.9
Japan	1	1.5
Portugal	1	1.5
Korea (Republic of)	3	4.4
Spain	2	2.9
Sweden	1	1.5
Switzerland	7	10.3
Taiwan	1	1.5
Türkiye	1	1.5
United Kingdom	11	16.2
United States of America	22	32.1

The patient and cross‐disciplinary expert surveys were launched on November 20, 2024. Multiple reminders were distributed to participants throughout the survey period to encourage engagement. The patient survey concluded on March 13, 2025, followed by the closure of the cross‐disciplinary expert survey on March 17, 2025. Participation was voluntary and uncompensated. The surveys were administered electronically via Microsoft Forms (Redmond, WA, USA). Informed consent was obtained as the first survey question; participants who did not provide consent were automatically excluded. All data were collected, stored, and processed anonymously.

### 2.4 Agreement Definition

While no universally accepted method exists for defining consensus in survey studies (von der Gracht 2012), this study applied the following thresholds for 7‐point Likert scale questions:

- Strong consensus: achieved when > 95% of the experts “somewhat agreed,” “agreed,” or “strongly agreed” with the statement made, or alternatively when > 95% “somewhat disagreed,” “disagreed,” and “strongly disagreed”;
- Consensus: defined when either agreement or disagreement was greater than 75% and no more than 95%;
- No consensus: defined when either agreement or disagreement was 75% or less.

Similarly, for multiple‐choice items that allowed the selection of one or multiple applicable statements (items 3, 10, and 14), strong consensus for any single statement was defined as selection by more than 95% of respondents; consensus was defined as selection by more than 75% and no more than 95%, and no consensus as ≤ 75% (Diamond et al. 2014). If consensus was not reached, the items were brought to the in‐person GCCG Workshop in Boston, where participants engaged in focused discussions and revised the items for reconsideration.

### 2.5 Statistical Analysis

Each question response was individually analyzed using descriptive statistics, with results expressed as percentages. The degree of agreement for each question was further detailed using the median score and interquartile range (IQR), adhering to the RAND guidelines (Khodyakov et al. 2023).

In the graphical representations, the percentage of agreement (%) was calculated by combining the responses “somewhat agree,” “agree,” and “strongly agree.” If the combined percentage of “somewhat disagree,” “disagree,” and “strongly disagree” was higher, that percentage was reported instead, with explanations provided in the figure footnotes. Summary graphs showing the median and IQR were developed in accordance with the RAND guidelines and included in the Figures S1–S6 (Khodyakov et al. 2023).

All analyses were conducted using Microsoft Forms, STATA v18, and GraphPad Prism v10.

## 3 Results

Of the 68 patients contacted, 41 accessed the survey and all of them provided consent and completed the questionnaire, resulting in a response rate of 60.3%. Of the 68 cross‐disciplinary experts contacted, 21 accessed the survey and all of them provided consent and completed the questionnaire, resulting in a response rate of 30.9%.

The surveys revealed that 15 statements across nine items achieved consensus or strong consensus among patient participants, while 15 statements from eight items reached similar levels of consensus among cross‐disciplinary experts.

### 3.1 Planning Phase

#### 3.1.1 Use of CBCT Scans

A consensus was reached about the use of CBCT scans for implant planning (92.7% for patient survey and 90.5% for cross‐disciplinary expert survey).

Regarding radiation exposure from CBCT scans, 36.6% of patients expressed concern, while 63.4% did not. Among cross‐disciplinary experts, 57.1% had concerns, whereas 42.9% did not.

#### 3.1.2 Preference for Removable vs. Fixed Full‐Arch Prosthesis

Among patients, 82.9% (*n* = 34) preferred a fixed maxillary full‐arch prosthesis over a removable one; four patients preferred a removable option, and three patients had no preference.

In the cross‐disciplinary expert group, 76.2% (*n* = 16) favored a fixed maxillary full‐arch prosthesis; 1 expert preferred a removable option, and 4 experts had no preference. Thus, a consensus favoring fixed prosthesis for both surveys was reached in both groups.

#### 3.1.3 Major Treatment Outcomes

We asked patients and cross‐disciplinary experts to identify the treatment outcomes they considered most important. Patients prioritized esthetics (95.1%, strong consensus), comfort (73.2%), and function (70.7%).

The cross‐disciplinary expert survey revealed that chewing function (90.5%, consensus), esthetics (85.7%, consensus), and easiness for cleaning (81.0%, consensus) were the primary outcomes of concern.

#### 3.1.4 Distal Extension Considerations for Full‐Arch Restorations

For a maxillary fixed full‐arch implant‐supported prosthesis, 63.4% of patients preferred molar extension, 12.2% found it acceptable to have premolars but not molars, and 24.4% had no preference. Among cross‐disciplinary experts, 71.4% preferred molar extension, 14.3% found premolars without molars acceptable, and another 14.3% had no preference.

#### 3.1.5 Preference for Provisional Prosthesis

Regarding the need of a provisional prosthesis, 58.5% of patients preferred to have a fixed provisional prosthesis; 17.1% of them preferred to have a removable provisional prosthesis, while 24.4% of them did not care if they had a provisional prosthesis. Among cross‐disciplinary experts, 47.6% of them preferred to have a fixed provisional prosthesis; 19.1% of them preferred to have a removable provisional prosthesis, while 33.3% of them did not care if they had a provisional prosthesis.

### 3.2 Treatment Procedures

#### 3.2.1 Hard Tissue Augmentation

When asked whether the need for a bone augmentation procedure along with its associated surgery and cost would influence their preference between a removable and fixed prosthesis, 80.5% of patients and 85.7% of cross‐disciplinary experts indicated they would still be open to undergoing the procedure for a fixed prosthesis. As a result, consensus was reached in both surveys.

#### 3.2.2 Preferred Impression Techniques

For the impression or scan method for the fabrication of the definitive prosthesis, 41.5% of patients preferred a digital scan; the other 58.5% of them did not have a preference. None of them preferred a conventional impression. Among cross‐disciplinary experts, 42.9% of them preferred a digital scan, 4.8% of them preferred a conventional impression; the other 52.3% of them did not have a preference.

### 3.3 Maintenance Care

#### 3.3.1 Frequency for Regular Check‐Ups

Regarding the frequency of regular check‐ups during the first year, 19.5% of patients preferred visits every 3 months, 39.0% preferred every 6 months, and 41.5% preferred annual visits. Among cross‐disciplinary experts, 57.1% favored check‐ups every 3 months, 19.0% preferred every 6 months, and 23.9% preferred once a year.

#### 3.3.2 Home Care and Occlusal Guards

For home care regimens, the patient survey indicated that toothbrushes (87.8%, consensus), water flossers (70.7%), and interdental brushes (41.5%) were the top choices for cleaning full‐arch prostheses at home. Similarly, the cross‐disciplinary expert survey identified toothbrushes (85.7%, consensus), interdental brushes (71.4%), and water flossers (57.1%) as the most preferred tools.

Regarding the use of occlusal guards, 65.9% of patients reported wearing them daily, 12.2% occasionally, and 21.9% rarely. Among cross‐disciplinary experts, 66.7% reported daily use, 19.0% occasional use, and 14.3% infrequent use.

#### 3.3.3 Potential Complications

For a full‐arch implant‐supported prosthesis, patients identified their top three concerns of potential complications as the risk of implant failure (58.5%), pain and swelling (48.8%), and the risk of prosthesis failure (39.0%). In the cross‐disciplinary expert survey, the top concerns were the risk of implant failure (71.4%), increased difficulty of cleaning (71.4%), and the risk of prosthesis failure (42.9%).

#### 3.3.4 Patient Communication

When asked about communication between patients and clinicians, 73.2% of patients felt very well‐informed about what to expect and the longevity of dental implants; 24.4% felt somewhat informed, and 2.4% felt not well‐informed. Similarly, 71.4% of cross‐disciplinary experts reported feeling very well‐informed, while 28.6% felt somewhat informed. None of the cross‐disciplinary experts reported feeling not well‐informed.

#### 3.3.5 Life Expectancy for Dental Implants

When asked about the expected lifespan of dental implants, 92.7% of patients anticipated their implants would last more than 10 years, while the remaining 7.3% expected a lifespan of 5–10 years. Notably, none of the patients expected their implants to last fewer than 5 years (strong consensus). Similarly, 90.5% of cross‐disciplinary experts anticipated a lifespan > 10 years, and 9.5% expected 5–10 years, with none expecting < 5 years (strong consensus). These responses reflect a clear consensus in both surveys regarding the expected longevity of dental implants.

### 3.4 Fundamental Outcomes to be Included in Future Studies

#### 3.4.1 Future Research on Maxillary Full‐Arch Rehabilitation With Dental Implants

**FIGURE 3:**
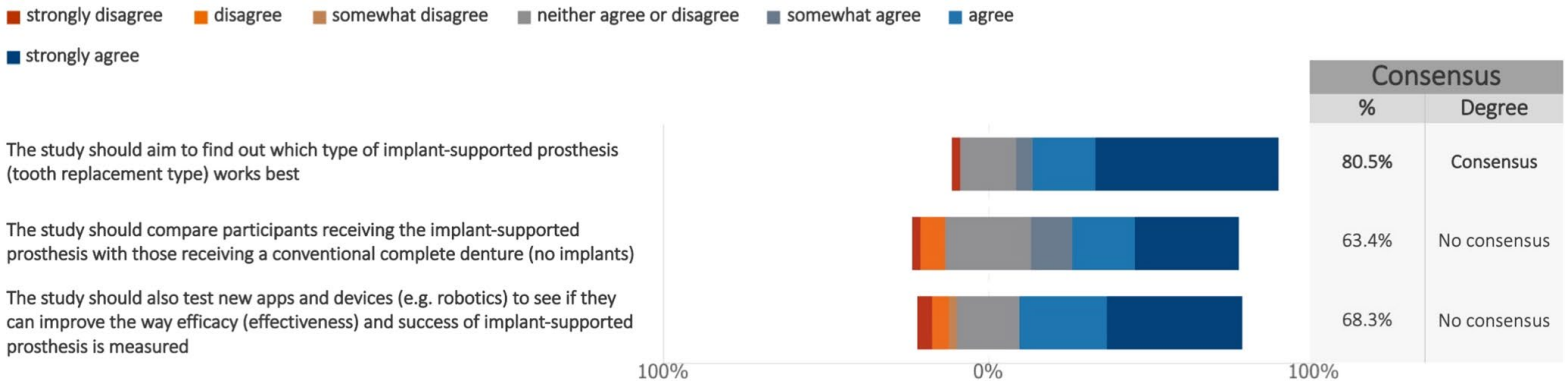
Overall goals and structure of future studies (professional investigations)—from the patient survey. The following questions ask about the overall goals and structure of your ideal study (to improve patient care, information, and treatment results).

**FIGURE 4:**
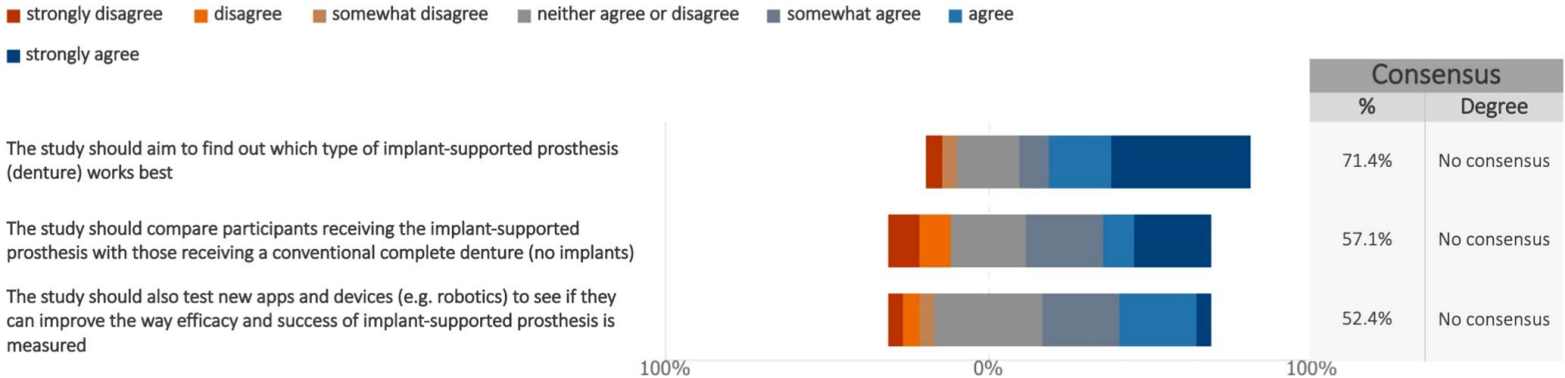
Overall goals and structure of future studies—from the cross‐disciplinary expert survey. The following questions ask about the overall goals and structure of your ideal study (to improve patient care, information, and treatment results).

When asked about the outcomes to be considered in future studies on maxillary full‐arch rehabilitation with dental implants, consensus was reached among patients (Figure 3, Figure S1) regarding the investigation of which type of implant‐supported prosthesis works best (median: 7; IQR: 5.5–7). However, this consensus was not shared by cross‐disciplinary experts (Figure 4, Figure S2). Additionally, consensus was not achieved in either group on comparing outcomes between implant‐supported prostheses and conventional complete dentures, or on the use of new technologies to enhance the efficacy and effectiveness of implant treatment.

#### 3.4.2 Relevant Patient‐Reported Outcomes (PROs)

**FIGURE 5:**
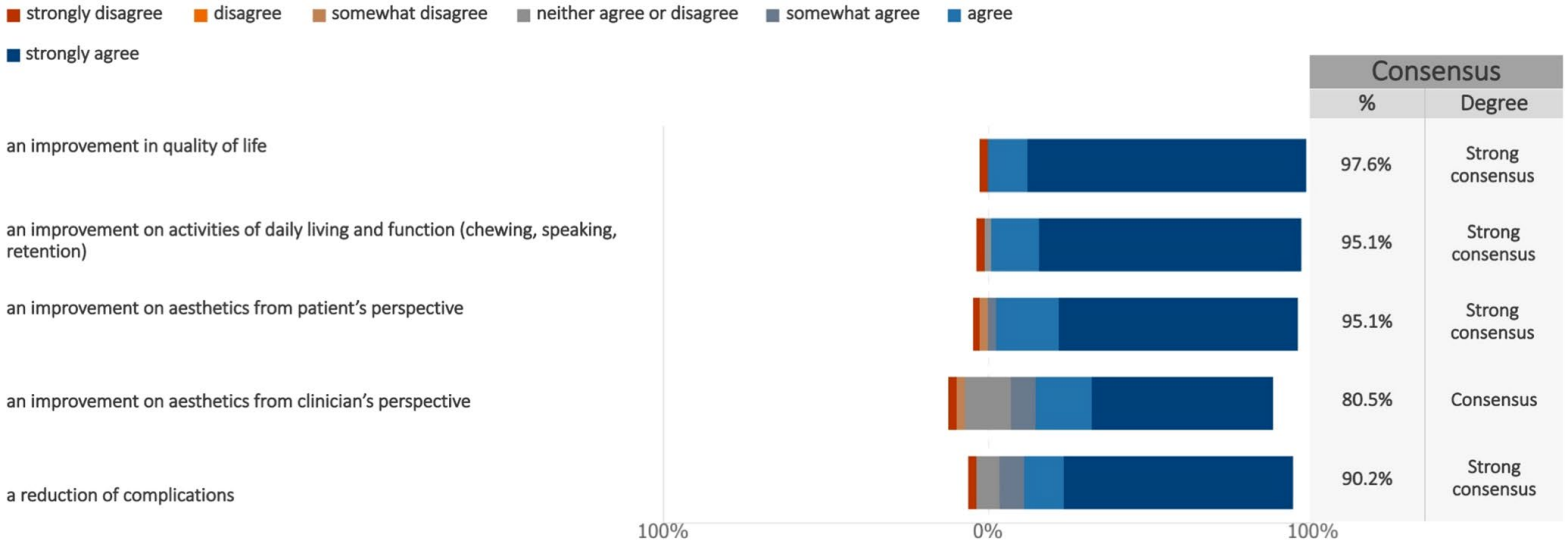
Measuring the effect of the new treatment—Part 1—from the patient survey. The following statements suggest ways to measure the results of implant‐supported prosthesis (tooth replacements). Please indicate whether you agree that these should be used to show the overall success of the study.

When asked about the relevant PROs to measure the outcomes of implant‐supported prostheses in future research, the patient survey (Figure 5, Figure S3) showed strong consensus on four key PROs: improvement in quality of life (median: 7; IQR: 7–7), enhancement of daily activities and function (median: 7; IQR: 6–7), improved esthetics from the patient's perspective (median: 7; IQR: 6–7), and reduction of complications (median: 7; IQR: 6–7). Additionally, consensus was achieved on one measure: improved esthetics from the clinician's perspective (median: 7; IQR: 5–7).

**FIGURE 6:**
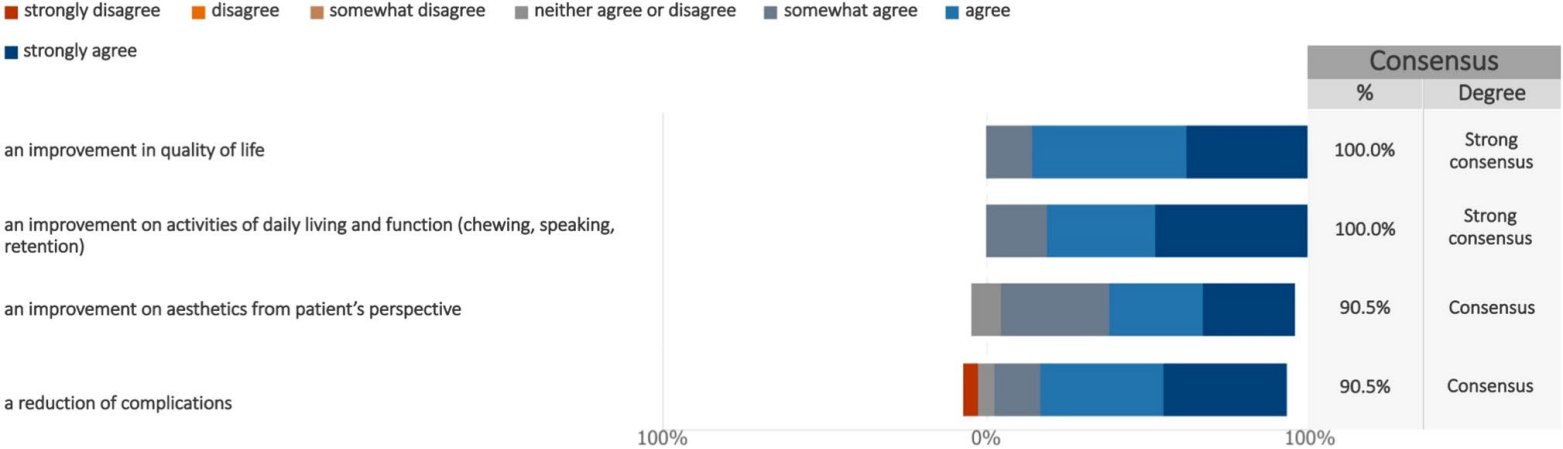
Measuring the effect of the new treatment—Part 1—from the cross‐disciplinary expert survey. The following statements suggest ways to measure the results of implant‐supported prosthesis (dentures). Please indicate whether you agree that these should be used to show the overall success of the study.

Similarly, the cross‐disciplinary expert survey (Figure 6, Figure S4) showed strong consensus on two PROs: improvement in quality of life (median: 6; IQR: 5–7) and enhancement of daily activities and function (median: 7; IQR: 6–7). Consensus was also reached on two additional PROs: improved esthetics from the patient's perspective (median: 7; IQR: 5–7) and reduction of complications (median: 6; IQR: 5.5–7).

#### 3.4.3 Difficulty of Procedures and Treatment Cost‐Effectiveness

**FIGURE 7:**
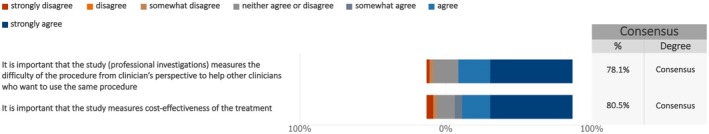
Measuring the effect of the new treatment—Part 2—from the patient survey. The following statements suggest ways to measure the results of implant‐supported prosthesis (dentures). Please indicate whether you agree that these should be used to show the overall success of the study.

**FIGURE 8:**
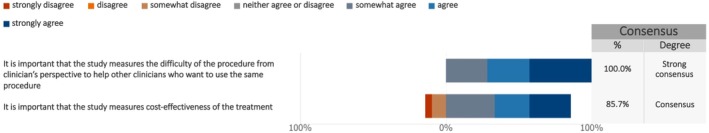
Measuring the effect of the new treatment—Part 2—from the cross‐disciplinary expert survey. The following statements suggest ways to measure the results of implant‐supported prosthesis (dentures). Please indicate whether you agree that these should be used to show the overall success of the study.

When asked about the appropriate ways to measure the difficulty of procedures and treatment cost‐effectiveness in future research, the patient survey (Figure 7, Figure S5) showed consensus on both statements: the importance of evaluating procedural difficulty from the clinician's perspective to inform other practitioners (median: 7; IQR: 6–7) and the importance of assessing the cost‐effectiveness of the treatment (median: 7; IQR: 5.5–7). Similarly, the cross‐disciplinary expert survey (Figure 8, Figure S6) showed strong consensus on the first statement (median: 6; IQR: 5–7) and consensus on the second statement (median: 6; IQR: 5–7).

## 4 Discussion

This report aimed to collect insights from patients and cross‐disciplinary experts in implant dentistry to guide and inform a structured consensus process during the first GCCG Workshop, focusing on treatment practices and outcomes for managing the edentulous maxilla.

### 4.1 Survey Participation

To explore global trends and developments in edentulous maxilla rehabilitation, 68 patients and 68 cross‐disciplinary experts were intentionally selected to represent a broad geographical distribution. However, due to the anonymity of responses, the actual extent of global representation could not be confirmed. The patient response rate was 60.3%, which is acceptable for survey‐based research (Story and Tait 2019). Conversely, the response rate among cross‐disciplinary experts was lower at 30.9%, indicating suboptimal participation. The low response rate may be attributed to several factors, including the length or complexity of the survey, survey fatigue, the anonymous nature of the survey reducing a sense of obligation to respond, and the absence of incentives (Artino Jr. et al. 2022; O'Reilly‐Shah 2017).

### 4.2 Treatment Planning

#### 4.2.1 Use of CBCT Scans

A consensus was reached regarding the use of CBCT scans for implant planning. This finding aligns with the American Academy of Periodontology (AAP) Best Evidence Consensus Statement on Selected Oral Applications for CBCT published in 2017, which supports the use of CBCT in the surgical management of dental implants (Mandelaris et al. 2017). CBCT imaging is valuable for assessing regional anatomy relevant to implant placement and offers clinicians improved accuracy in both presurgical planning and surgical execution.

However, no consensus emerged concerning potential concerns related to radiation exposure from CBCT scans. Currently, the long‐term effects of cumulative CBCT radiation remain uncertain (De Vos et al. 2009; Mandelaris et al. 2017). When susceptible tissues are properly shielded and the field of view is limited to the area of interest, the radiation risk is considered low (Mandelaris et al. 2017). Despite this, minimizing patient exposure remains essential, and clinicians must adhere to established radiation safety principles. Importantly, a recent systematic review found that using low‐dose CBCT protocols does not compromise objective image quality during any stage of implant therapy (Kaaber et al. 2024), suggesting a viable strategy for balancing diagnostic value and radiation safety.

#### 4.2.2 Preference for Removable Versus Fixed Full‐Arch Prosthesis

A consensus was achieved among both patients and cross‐disciplinary experts in favor of a maxillary fixed full‐arch implant‐supported prosthesis over a removable alternative. A systematic review reported that a fixed full‐arch implant‐supported prosthesis might provide a better implant and prosthesis survival rate (Lan et al. 2025). Additionally, a fixed prosthesis could provide better function‐related outcomes than removable options (Messias et al. 2023); however, removable implant‐supported prostheses have been associated with reduced marginal bone loss (Lan et al. 2025) and a lower risk of peri‐implantitis (Messias et al. 2023). It has been reported that although patients had similar functional and social improvements with both types of restorations, treatment with fixed implant‐supported prostheses was additionally perceived as a form of “normalization,” with patients viewing these restorations as closely resembling natural teeth (Kashbour et al. 2015). Furthermore, a recent systematic review comparing implant‐supported overdentures with fixed full‐arch prostheses suggested that removable options may be easier for maintaining oral hygiene (Yao et al. 2018). However, the results were inconsistent, and the review (Yao et al. 2018) included patients with edentulous mandibles, which were not considered in the present surveys.

#### 4.2.3 Major Treatment Outcomes

In the patient survey, a strong consensus identified esthetics as the most important treatment outcome. While comfort and function did not reach the consensus threshold, a majority agreement ranked them as the second (73.2%) and third (70.7%) most important outcomes, respectively. Conversely, in the cross‐disciplinary expert survey, chewing function was identified as the highest‐priority outcome, followed by esthetics and ease of cleaning. All three outcomes reached the consensus level among experts. These findings suggest that patients may place greater emphasis on esthetics, whereas cross‐disciplinary experts tend to prioritize function. It should be noted, however, that patients tend to be less critical when it comes to esthetics. For instance, a recent systematic review with meta‐analysis on soft‐tissue augmentation around implant sites found that although clinicians may observe a slight esthetic advantage with autogenous grafts over soft‐tissue substitutes, this difference is typically not noticeable to patients (Ramanauskaite et al. 2025).

A systematic review analyzing PROs for fixed full‐arch implant‐supported prostheses and IODs identified chewing function, speech, overall satisfaction, and esthetics as the most frequently reported domains (Yao et al. 2018). However, the literature remains inconclusive regarding whether fixed prostheses offer superior outcomes compared to IODs in these areas (Brennan et al. 2010; Heydecke et al. 2003; Yao et al. 2018). Nonetheless, these aspects were integrated into the “functional and esthetic domain” defined by the recent multi‐stakeholder initiative—the Implant Dentistry Core Outcome Set and Measurements (ID‐COSM), which aims to establish an internationally recognized core outcome set in implant dentistry. This initiative identified 13 core outcomes, grouped into nine domains: function, surgical complications, loss of tissue health, adverse device events, implant/restoration survival and success, implant loss/failure/fracture, quality of life, overall satisfaction, and maintenance effort. The ID‐COSM also employed the methodology to gather broad, consensus‐driven input (Sanz et al. 2023).

#### 4.2.4 Distal Extension Considerations for Full‐Arch Restorations

Although consensus was not achieved, both patients and cross‐disciplinary experts reached a majority agreement favoring molar extension in the maxillary fixed full‐arch implant‐supported prosthesis. However, the evidence supporting this concept remains limited. Although a 5‐year randomized controlled trial recommended first molar occlusion for its biomechanical advantages (Toia et al. 2025), the absence of consensus highlights the need for further clinical trials to substantiate this recommendation.

#### 4.2.5 Preference for Provisional Prosthesis

No consensus was reached regarding the preferred type of provisional prosthesis. However, a majority of patients indicated a preference for a fixed provisional prosthesis over a removable option. Although immediate fixed provisionalization has been reported to offer advantages such as improved patient comfort, enhanced function during the implant healing period, and fewer postoperative adjustments compared to conventional dentures (Santosa 2007), there remains a lack of direct evidence comparing the clinical outcomes of fixed versus removable full‐arch provisional prostheses. Consequently, the selection of a provisional prosthesis should be based on individual esthetic expectations, functional requirements, anticipated duration of use, and ease of fabrication (Cho et al. 2007).

### 4.3 Treatment Procedures

#### 4.3.1 Hard Tissue Augmentation

A consensus was reached among both patients and cross‐disciplinary experts supporting the acceptance of bone augmentation procedures, including the associated surgical intervention, when required to achieve a fixed prosthesis. This finding is likely attributable to the high success rate and predictability of bone augmentation techniques (Aghaloo et al. 2016; Calciolari et al. 2023; Chiapasco et al. 2009). However, despite being generally safe and predictable, bone augmentation procedures have been associated with complications such as barrier membrane exposure, infection, and soft tissue dehiscence (Calciolari et al. 2023; Garcia et al. 2018; Lim et al. 2018; Thoma et al. 2019; Urban et al. 2019). Therefore, clinicians should carefully consider these potential complications when planning hard tissue augmentation for implant site development.

#### 4.3.2 Preferred Impression Techniques

No consensus was reached among patients or cross‐disciplinary experts regarding preferred impression techniques. These findings contradict a recent systematic review and meta‐analysis (de Paris Matos et al. 2023), which reported that patients preferred and were more satisfied with digital scanning over conventional impression methods. The review highlighted that digital scanning caused less discomfort, particularly for patients sensitive to taste, nausea, or breathing difficulties (de Paris Matos et al. 2023). This discrepancy is due to differences in inclusion criteria, as the review excluded studies involving fully edentulous patients.

Conversely, neither digital nor conventional impressions achieved majority agreement. This result is consistent with the current evidence (Cai et al. 2024; Gehrke et al. 2024; Tallarico et al. 2019) that the accuracy is comparable between conventional impression techniques and intraoral scanning. Future studies should investigate factors that may influence the accuracy of intraoral scanning, such as scan body placement, shape, material composition, and color, the manufacturing system, scanning technique, number of implants, inter‐implant distance, angulation, and depth of existing implants, etc., in order to further optimize its precision (Gehrke et al. 2024; Revilla‐Leon et al. 2023a, 2023b).

### 4.4 Maintenance Care

#### 4.4.1 Frequency for Regular Check‐Ups

There was no consensus between patients and cross‐disciplinary experts regarding the recommended frequency of regular check‐ups. Interestingly, patients tended to favor annual or biannual visits over every 3 months, whereas cross‐disciplinary experts showed a preference for check‐ups every 3 months rather than every 6 months or annually. These findings are largely consistent with the European Federation of Periodontology S3 guideline, which, although emphasizing the importance of supportive peri‐implant care in minimizing the risk of peri‐implant diseases, does not specify an exact interval for such visits in patients with healthy peri‐implant tissues (Herrera et al. 2023).

It has been recommended that patients identified by their dentists as higher risk due to factors such as advanced age, limited ability to maintain oral hygiene, or the presence of biological or mechanical complications related to implant‐supported restorations should receive professional dental evaluations more frequently than the standard 6‐month interval (Bidra et al. 2016).

#### 4.4.2 Home Care and Occlusal Guards

In the patient survey, a consensus was reached on the preferred home care regimen: toothbrushes (87.8%) and water flossers (70.7%) were identified as the top choices for cleaning full‐arch prostheses. Although interdental brushes ranked third, they did not meet the consensus threshold. Similarly, cross‐disciplinary experts reached consensus on toothbrushes (85.7%) and interdental brushes (71.4%) as the top choices, with water flossers ranking third but falling short of the consensus level. This finding is consistent with the currently available evidence. A wide range of oral hygiene tools is available to support home care, including manual toothbrushes, powered brushes with counter‐rotational or sonic action, single‐tuft brushes, interdental brushes, dental floss, toothpaste, oral irrigators, and mouth rinses (Perussolo and Donos 2024). The choice and use of these aids should be tailored to the individual needs of each patient through appropriate professional instruction. However, current research indicates that there is no universally established protocol for oral hygiene practices around dental implants. The most effective type of hygiene aid, along with the optimal frequency and duration of use, remains uncertain for maintaining peri‐implant health and minimizing the risk of disease recurrence (Herrera et al. 2023). It is important to provide individualized home care instructions tailored to each patient, taking into account factors such as implant design, ease of access for cleaning, and the patient's manual dexterity (Armitage and Xenoudi 2016; Todescan et al. 2012). For instance, a smaller‐diameter interdental brush can be used to clean areas with limited access. In cases where implant‐supported restorations present extremely restricted access for hygiene, the use of a stiff‐ended dental floss threader, combined with the “criss‐cross” technique, can effectively support plaque removal during home care. Patients should receive chairside instruction on how to properly insert the floss threader through the embrasure space beneath the implant‐supported restoration. Once inserted, the floss should be wrapped around the restoration and gently moved back and forth along the surfaces of each implant to ensure thorough cleaning.

Regarding the use of occlusal guards, no consensus was achieved among either patients or cross‐disciplinary experts. Nevertheless, both groups showed a majority agreement that occlusal guards should be used daily. Although implants placed in probable bruxers carry a significantly higher risk of failure compared to those placed in non‐bruxers (Haggman‐Henrikson et al. 2024), high‐quality evidence supporting the routine use of appliances in bruxers undergoing dental implant rehabilitation remains limited (Haggman‐Henrikson et al. 2024; Mesko et al. 2014; Sutthiboonyapan and Wang 2019).

#### 4.4.3 Potential Complications

While no consensus was achieved among either patients or cross‐disciplinary experts regarding potential complications, the patient survey revealed a majority agreement identifying implant failure as the primary concern. Correspondingly, cross‐disciplinary experts reached majority agreement on implant failure and increased difficulty in cleaning as the most significant anticipated complications. Although implant failure (Goodacre et al. 2003; Goodacre et al. 1999) and increased difficulty in cleaning (Serino and Strom 2009) have been identified as potential complications for partially edentulous patients receiving dental implants, the literature specifically addressing complications related to full‐arch implant‐supported prostheses is limited (Papaspyridakos et al. 2012; Papaspyridakos et al. 2025) and lacks consensus.

#### 4.4.4 Patient Communication

Although no consensus was reached in either the patient or cross‐disciplinary expert surveys, both groups demonstrated a majority agreement indicating they felt well‐informed about treatment expectations and the anticipated longevity of dental implants. These findings align with the collaborative model known as shared decision making, which emphasizes equal authority and mutual participation between patient and clinician, treating the patient as an active partner in care (Shutzberg 2021). Supporting this, a recent survey of 96 patients with periodontitis found that patients prefer to be well‐informed and involved in decision‐making with healthcare professionals following their diagnosis (Jung et al. 2025). This finding suggests the importance of providing patients with clear, accurate, and evidence‐based information when considering dental implant treatment. Ensuring that patients are fully informed fosters realistic expectations, which in turn contributes to higher levels of satisfaction with the outcomes of implant therapy (Colvin et al. 2019; McCrea 2017).

#### 4.4.5 Life Expectancy for Dental Implants

Although no consensus was reached in either the patient or cross‐disciplinary expert surveys, both groups showed majority agreement that they felt well‐informed about treatment expectations and the anticipated longevity of dental implants. Additionally, a strong consensus was reached across both groups that none expected dental implants to last < 5 years. Although implant survival rates have been reported as high, with 100% for fixed complete prostheses and 95.7% for overdentures, the corresponding success rates were relatively lower, with 63.8% for fixed complete prostheses and 78.6% for overdentures over a 7‐year follow‐up period (Brocard et al. 2000). Additionally, while a questionnaire‐based study reported a 70% rate of post‐surgical satisfaction among patients who received dental implants; however, the majority of participants (74.1%) lacked knowledge about peri‐implant pathology at the time of treatment (Insua et al. 2017). These findings highlight the need for clinicians to develop standardized educational materials and decision aids, such as brochures, to inform patients about risk factors and clinical indicators associated with peri‐implant disease, thereby supporting its prevention.

### 4.5 Fundamental Outcomes to be Included in Future Studies

#### 4.5.1 Future Research on Maxillary Full‐Arch Rehabilitation With Dental Implants

For future research on maxillary full‐arch rehabilitation with dental implants, a consensus was reached among patients supporting the investigation of which type of implant‐supported prosthesis yields the best outcomes. However, this consensus was not shared by cross‐disciplinary experts. Given the multitude of factors influencing clinical decision‐making in the rehabilitation of the edentulous maxilla, future research should focus on evaluating variables such as esthetic demands, type of prosthetic support, degree of alveolar resorption and inter‐arch space, number and distribution of implants, implant positioning, and overall treatment cost (Jivraj et al. 2006).

#### 4.5.2 Relevant Patient‐Reported Outcomes (PROs)

The patient survey showed strong consensus on four key PROs in future research: improvement in quality of life, enhancement of daily activities and function, improved esthetics from the patient's perspective, and reduction of complications. Additionally, consensus was achieved on one clinician‐centered outcome: improved esthetics from the clinician's perspective. Notably, patients showed stronger agreement regarding the importance of esthetics from their own perspective than from that of clinicians.

Similarly, the cross‐disciplinary expert survey showed strong consensus on two PROs: improvement in quality of life and enhancement of daily activities and function. Consensus was also reached on two additional PROs: improved esthetics from the patient's perspective and reduction of complications. A recent systematic review on patients' expectations regarding dental implants identified esthetics and function as key attributes influencing their treatment preferences (Yao et al. 2014).

These findings align with previous systematic reviews highlighting the increasing importance of PROs in implant dentistry (De Bruyn et al. 2015; Feine et al. 2018; Yao et al. 2018). Evidence suggests that incorporating PROMs into clinical practice enhances patient‐clinician communication, which can, in turn, improve treatment processes and outcomes (Nelson et al. 2015; Sutherland and Till 1993). PROMs may also serve as a tool for patients to articulate their values and concerns, thereby fostering shared decision‐making and greater patient engagement in their care (Feldman‐Stewart and Brundage 2009; Santana and Feeny 2014).

#### 4.5.3 Difficulty of Procedures and Treatment Cost‐Effectiveness

Both the patient and cross‐disciplinary expert surveys demonstrated strong consensus on two statements in future research: (1) the importance of evaluating procedural difficulty from the clinician's perspective to inform other practitioners and (2) the importance of assessing the cost‐effectiveness of the treatment.

A recent systematic review (Thoma et al. 2026) noted that subjective ClinROs such as perceived ease or difficulty of procedures are rarely reported in the literature. This is likely due to the traditional emphasis on objective outcomes, with limited attention given to clinician preferences and perspectives. Moreover, many studies treating edentulous patients involved highly experienced clinicians, whose skill levels may not reflect the daily clinical realities faced by general practitioners.

Additional support for the importance of clinician preferences comes from a consensus report on extraction socket management and implant timing (Tonetti et al. 2019), which emphasizes that treatment decisions are often influenced by clinicians' own experience. Capturing subjective ClinROs such as procedural ease or complexity could provide meaningful guidance for less experienced clinicians, helping them select appropriate treatment strategies based on practical considerations (Cosyn et al. 2023; Thoma et al. 2024).

Regarding cost‐effectiveness, current evidence suggests that for the replacement of multiple teeth, dental implants, whether supporting fixed or removable prostheses, are associated with higher initial costs but greater improvements in oral health‐related quality of life compared to alternative treatments (Vogel et al. 2013). The EAO consensus conference on the economic evaluation of implant‐supported prostheses reported that mandibular overdentures retained by two or four implants significantly enhanced oral health‐related quality of life, albeit at a higher cost than conventional complete dentures (Beikler and Flemmig 2015). Although evidence on the cost‐effectiveness of maxillary full‐arch rehabilitation with dental implants remains limited, a recently published randomized controlled trial indicated that, from a short‐term perspective, implant‐supported overdentures in the edentulous maxilla incurred comparable maintenance and repair costs over the first year and remained the least costly treatment option (Ghiasi et al. 2022).

The limited inclusion of outcomes related to procedural difficulty and cost effectiveness in studies on edentulous maxilla rehabilitation may reflect the absence of a core outcome set or the poor uptake of existing ones (Tonetti, Heitz‐Mayfield, et al. 2023). A core outcome set is a standardized set of essential outcomes that should be routinely measured and reported (Clarke 2007). In response, the ID‐COSM was recently introduced (Tonetti, Heitz‐Mayfield, et al. 2023) to further standardize the reporting requirements.

The adoption of such standardized outcome measures is expected to improve consistency and comparability across studies and enhance patient care. Importantly, ID‐COSM recommendations do not preclude the inclusion of additional outcomes. Rather, they ensure that a minimum set of essential outcomes is consistently captured, while allowing flexibility to address study‐specific objectives (Williamson et al. 2017). The present study builds upon these initiatives by contributing a more focused effort to define both PROMs and ClinROs specific to the rehabilitation of the edentulous maxilla.

### 4.6 Limitations and Future Research Directions

Several limitations of this study should be acknowledged. First, the response rates were relatively low (60.3% for the patient survey and 30.9% for the cross‐disciplinary expert survey), which may limit the representativeness of the sample and affect the generalizability of the findings. Second, there is a potential for selection bias in the recruitment of both patients and cross‐disciplinary experts, given the involvement of the GCCG Scientific Task Force in the engagement process. This could have influenced the perspectives captured. Third, although efforts were made to design a comprehensive survey process that included multi‐domain stakeholder engagement, there remains a lack of established frameworks guiding the integration of patients and cross‐disciplinary experts in survey studies within dental research. As such, the findings of this study should be interpreted with caution, considering these methodological constraints.

After reviewing the survey results, we identified several gaps in the existing literature related to treatment planning and clinical decision‐making for edentulous maxilla rehabilitation. Future research should address key areas such as the establishment of optimal follow‐up and maintenance protocols, the development of validated clinician‐reported outcome measures, and the creation of guidelines to support effective patient communication and management of expectations. Additionally, building upon the foundational efforts of this study, it will be important to further develop structured frameworks for the meaningful integration of patients and cross‐disciplinary experts in survey studies within dental research.

## 5 Conclusion

This study collected feedback from patients and cross‐disciplinary experts to aid in the creation of a clinical practice guideline for managing the edentulous maxilla. The surveys revealed that 15 statements across nine items achieved consensus or strong consensus among patient participants, while 15 statements from eight items reached similar levels of consensus among cross‐disciplinary experts. Items that did not reach consensus offered insight into real‐world implant treatment practices, highlighted gaps in the existing literature, and revealed discrepancies between patient perspectives, cross‐disciplinary expert opinions, and the current body of evidence. While the results offer valuable preliminary insights, they reflect the views of a limited group of respondents and should not be interpreted as a formal consensus.

## Author Contributions

**Guo‐Hao Lin:** methodology, validation, formal analysis, investigation, data curation, writing – original draft. **Giulia Brunello:** methodology, software, data curation, investigation, validation, formal analysis, visualization, writing – original draft. **Ronald E. Jung:** conceptualization, supervision, writing – review and editing, project administration, resources. **Ina Kopp:** conceptualization, methodology, data curation, writing – review and editing, visualization, supervision. **Frank Schwarz:** conceptualization, resources, project administration, writing – review and editing, supervision. **Hom‐Lay Wang:** conceptualization, supervision, project administration, resources, writing – review and editing. **Franz J. Strauss:** methodology, software, data curation, investigation, validation, formal analysis, visualization, writing – original draft.

## Ethics Statement

The protocol was notified to and approved by the Ethical Committee of the University of Düsseldorf (Protocol no. 2024‐2973_1).

## Conflicts of Interest

The authors declare no conflicts of interest.

## Supporting information


**Appendix S1:** clr70023‐sup‐0001‐AppendixS1.pdf.

**Appendix S2:** clr70023‐sup‐0002‐AppendixS2.pdf.

**Data S1:** clr70023‐sup‐0003‐Figures.docx.

## Data Availability

The data that support the findings of this study are available from the corresponding author upon reasonable request.
