# Supplementary material for: 1st Global Consensus for Clinical Guidelines for the Rehabilitation of the Edentulous Maxilla: Patient and Cross‐Disciplinary Expert Single‐Round Surveys
Source: Clin Oral Implants Res. 2026 Feb 24;37(Suppl 30):S188–203. doi: 10.1111/clr.70023 (PMC12930135; doi:10.1111/clr.70023)
Supplement: Supplementary file 1 — Appendix S1: clr70023‐sup‐0001‐AppendixS1.pdf. [file CLR-37-S188-s003.pdf]

Patient Survey – 1st Global Consensus for Clinical Guidelines 2025

* Required

1. If you click on "yes", it means that you consent to participate in the survey, otherwise it ends here. * *

   ◯ Yes

   ◯ No

2. Do you prefer to have a removable or fixed maxillary full-arch prosthesis (i.e a replacement of your upper teeth that is removable by you or fixed in place)? *

   ◯ Removable

   ◯ Fixed

   ◯ No preference

3. What are the major outcomes you focus on in your treatment that you prefer to see? *

   Please choose five of the following as your highest priority.

   Please select at most 5 options.

   ☐ Aesthetics (appearance)

   ☐ Chewing function (ease of chewing)

   ☐ Phonetics (capability to pronounce the word)

   ☐ Easiness for cleaning

   ☐ Number of surgical interventions

   ☐ Comfort

   ☐ Cost

   ☐ Total treatment time (from the beginning to the end)

4. Do you normally receive a 3D X-ray scan (Cone Beam Computed Tomography / CBCT scan) before implant placement? *

   In this question we do not refer to 2D X-ray (panoramic radiograph), but only to 3D X-ray scan.

   ◯ Yes. I normally receive a CBCT scan before implant treatment.

   ◯ No. I normally do not receive a CBCT scan before implant treatment.

5. Are you concerned about CBCT scans and associated radiation doses, particularly when multiple scans are needed during the course of treatment? *

   ◯ Yes

   ◯ No

6. For full-arch restorations, do you prefer the prosthesis to extend to the molar region (i.e include replacement of the larger back teeth), or is extension to the premolar (bicuspid) region acceptable (i.e just replacement of the smaller side teeth)? *

- ◯ Prefer to extend to molars *(12-14 teeth in the upper jaw)*
- ◯ Premolar/bicuspid region is fine *(10 teeth in the upper jaw)*
- ◯ No preference

7. If a bone augmentation procedure (i.e. bone grafting/regeneration) is required for implant placement to have a fixed prosthesis with the inherent additional surgery and cost, does this change your preference for receiving a removable prosthesis versus a fixed prosthesis? *

- ◯ Yes. I will prefer to avoid bone augmentation procedure; removable option is fine with me if I can avoid this procedure.
- ◯ No. I will be open to receive bone augmentation procedure to achieve the goal of a fixed prosthesis.

8. Do you prefer to receive a provisional prosthesis (i.e a temporary tooth replacement after implant placement which is either fixed or removable) on the same day of the surgery, or is it acceptable not to have a provisional prosthesis? *

- ◯ Yes. I will prefer to have a fixed provisional prosthesis on the same day.
- ◯ Yes. I will prefer to have a removable provisional prosthesis on the same day.
- ◯ No. I don't care if I have a provisional prosthesis on the same day or not.

9. After the delivery/completion of the full-arch implant-supported prosthesis in the upper jaw, how often do you prefer to come back for a regular check-up? *

- ◯ I will prefer to come back every 3 months for a regular check-up for the first year.
- ◯ I will prefer to come back every 6 months for a regular check-up for the first year.
- ◯ I will prefer to come back once a year for a regular check-up.

10. How do you plan to clean your full-arch prosthesis at home? *

Choose all the ones applicable to you.

- [ ] Brushing the implants with a toothbrush and/or toothpaste
- [ ] Flossing the implants
- [ ] Interdental brush
- [ ] Mouthwash / mouthrinse
- [ ] Rubber tip
- [ ] Water flosser / WaterPik
- [ ] Other

11. If you are offered an occlusal guard to wear at night (to protect your upper tooth replacement/prosthesis during sleep), will you be willing to wear it every day? *

- ◯ I will wear it every day as suggested.
- ◯ I will wear it from time to time due to comfort.
- ◯ I won't wear it often, or I'll deny occlusal guard.

12. How long do you expect your implants to last? (<5 years, 5-10 years, >10 years) *

- ◯ <5 years; I understand that implant treatment might not last forever.
- ◯ 5-10 years; I expect that implants can last for a while but not forever.
- ◯ >10 years; implant treatment is expected to last for a long period of time.

13. For the final impression process (for making your definitive tooth replacement/prosthesis), do you have a preference for a digital scan, a conventional scan (i.e traditional impression), or depending on the provider's preference (i.e your dentist's preferred method)? *

- ◯ I'll prefer a state-of-the-art digital impression.
- ◯ I'll prefer a conventional alginate impression.
- ◯ I don't have a preference.

14. What concerns or questions do you have about potential complications (i.e what could go wrong) related to your full-arch prosthesis treatment? *

Please choose three of the following as your highest priority.

Please select 3 options.

- ☐ Pain/swelling from the surgical procedure(s)
- ☐ Transitional stage without the final denture (i.e period between implant placement and getting the definitive tooth replacement/prosthesis)
- ☐ Need for taking many medications for post-surgery care
- ☐ Need for more frequent check-up appointments in the future
- ☐ Increased difficulty of cleaning
- ☐ Increased difficulty of speaking
- ☐ Risk of implant failure (such as problems with inflamation or loss of the implant)
- ☐ Risk of prosthesis failure (such as breakage)
- ☐ Possible change of the bite/ joint relation (i.e changes in the contact between upper and lower teeth leading to jaw joint problems)
- ☐ Other

15. How well-informed do you feel your doctor(s) communicated with you about what to expect and the longevity of your implant? *

- ◯ I feel very well-informed; my doctor(s) informed me that the implants and the prosthesis may need to be replaced at some point.
- ◯ I feel somewhat informed; my doctor(s) mentioned some information related to the implant/prosthesis longevity, but no details were provided.
- ◯ I feel not well-informed; my doctor(s) did not provide information related to the implant/prosthesis longevity throughout the treatment process.

16. **Overall goals and structure of future studies (professional investigations).** The following questions ask about the overall goals and structure of your ideal study (to improve patient care, information and treatment results). *

*On a scale of 1 to 7 (where 1 is "strongly disagree" and 7 is "strongly agree"), [**Make sure to slide the table all the way to the right to see all score levels, including "7, strongly agree."],** please indicate how much you agree with the following statements:*

	1 (strongly disagree)	2	3	4 (neutral)	5	6	7 (strongly agree)
The study should aim to find out which type of implant-supported prosthesis (tooth replacement type) works best	○	○	○	○	○	○	○
The study should compare participants receiving the implant-supported prosthesis with those receiving a conventional complete denture (no implants)	○	○	○	○	○	○	○
The study should also test new apps and devices (i.e techniques e.g. robotics) to see if they can improve the way efficacy (effectiveness) and success of implant-supported prosthesis is measured	○	○	○	○	○	○	○

17. **Measuring the effect of the new treatment - Part 1.** The following statements suggest ways to measure the results of implant-supported prosthesis (tooth replacements). Please indicate whether you agree that these should be used to show the overall success of the study. *

*On a scale of 1 to 7 (where 1 is "strongly disagree" and 7 is "strongly agree"), [**Make sure to slide the table all the way to the right to see all score levels, including "strongly agree."],** it is important that the overall success of the study is shown by:*

	1 (strongly disagree)	2	3	4 (neutral)	5	6	7 (strongly agree)
an improvement in quality of life	○	○	○	○	○	○	○
an improvement on activities of daily living and function (chewing, speaking, retention)	○	○	○	○	○	○	○
an improvement on aesthetics from patient's perspective	○	○	○	○	○	○	○
an improvement on aesthetics from clinician's perspective	○	○	○	○	○	○	○
a reduction of complications	○	○	○	○	○	○	○

18. **Measuring the effect of the new treatment - Part 2.** The following statements suggest ways to measure the results of implant-supported prosthesis (dentures). Please indicate whether you agree that these should be used to show the overall success of the study. *

*On a scale of 1 to 7 (where 1 is "strongly disagree" and 7 is "strongly agree"), please indicate how much you agree with the following statements [**Make sure to slide the table all the way to the right to see all score levels, including "7, strongly agree."]**:*

	1 (strongly disagree)	2	3	4 (neutral)	5	6	7 (strongly agree)
It is important that the study (professional investigations) measures the difficulty of the procedure from clinician's perspective to help other clinicians who want to use the same procedure	○	○	○	○	○	○	○
It is important that the study measures cost-effectiveness of the treatment	○	○	○	○	○	○	○

19. Are there any important results, outcomes, or aspects that you think should be measured in patients receiving an implant-supported prosthesis (implant-retained denture) that were not mentioned in the previous questions? *

This content is neither created nor endorsed by Microsoft. The data you submit will be sent to the form owner.

Microsoft Forms
