# Supplementary material for: 1st Global Consensus for Clinical Guidelines for the Rehabilitation of the Edentulous Maxilla: Patient and Cross‐Disciplinary Expert Single‐Round Surveys
Source: Clin Oral Implants Res. 2026 Feb 24;37(Suppl 30):S188–203. doi: 10.1111/clr.70023 (PMC12930135; doi:10.1111/clr.70023)
Supplement: Supplementary file 2 — Appendix S2: clr70023‐sup‐0002‐AppendixS2.pdf. [file CLR-37-S188-s001.pdf]

Stakeholder Survey – 1st Global Consensus for Clinical Guidelines 2025

* Required

1. If you click on "yes", it means that you consent to participate in the survey, otherwise it ends here. *

   ◯ Yes

   ◯ No

2. Do you prefer to have a removable or fixed maxillary full-arch prosthesis? *

   ◯ Removable

   ◯ Fixed

   ◯ No preference

3. What are the major outcomes you focus on in your treatment that you prefer to see? *
   Please choose five of the following as your highest priority.

   Please select 5 options.

   ☐ Aesthetics

   ☐ Chewing function

   ☐ Phonetics

   ☐ Easiness for cleaning

   ☐ Number of surgical interventions

   ☐ Comfort

   ☐ Cost

   ☐ Total treatment time (from the beginning to the end)

4. Do you normally receive a CBCT scan before implant placement? *

   ◯ Yes. I normally receive a CBCT scan before implant treatment.

   ◯ No. I normally do not receive a CBCT scan before implant treatment.

5. Are you concerned about Cone Beam CT scans and associated radiation doses, particularly when multiple scans are needed during the course of treatment? *

   ◯ Yes

   ◯ No

6. For full-arch restorations, do you prefer the prosthesis to extend to the molar region, or is extension to the premolar (bicuspid) region acceptable? *

   ◯ Prefer to extend to molars

   ◯ Premolar/bicuspid region is fine

   ◯ No preference

7. If a bone augmentation procedure (i.e the regeneration of the bone) is required for implant placement to have a fixed prosthesis with the inherent additional surgery and cost, does this change your preference for receiving a removable prosthesis versus a fixed prosthesis? *

   ◯ Yes. I will prefer to avoid bone augmentation procedure; removable option is fine with me if I can avoid this procedure.

   ◯ No. I will be open to receive bone augmentation procedure to achieve the goal of a fixed prosthesis.

8. Do you prefer to receive a provisional prosthesis (either a fixed or removable one; differentiate them) on the same day of the surgery, or is it acceptable not to have a provisional prosthesis? *

- ◯ Yes. I will prefer to have a fixed provisional prosthesis on the same day.
- ◯ Yes. I will prefer to have a removable provisional prosthesis on the same day.
- ◯ No. I don't care if I have a provisional prosthesis on the same day or not.

9. After the delivery/completion of the full-arch maxillary implant-supported prosthesis, how often do you prefer to come back for a regular check-up? *

- ◯ I will prefer to come back every 3 months for a regular check-up for the first year.
- ◯ I will prefer to come back every 6 months for a regular check-up for the first year.
- ◯ I will prefer to come back once a year for a regular check-up.

10. How do you plan to clean your full-arch prosthesis at home? *

Choose all the ones applicable to you.

- ☐ Brushing the implants with a toothbrush and/or toothpaste
- ☐ Flossing the implants
- ☐ Interdental brush
- ☐ Mouthwash / mouthrinse
- ☐ Rubber tip
- ☐ Water flosser / WaterPik
- ☐ Other

11. If you are offered an occlusal guard to wear at night, will you be willing to wear it every day? *

- ◯ I will wear it every day as suggested.
- ◯ I will wear it from time to time due to comfort.
- ◯ I won't wear it often, or I'll deny occlusal guard.

12. How long do you expect your implants to last? (<5 years, 5-10 years, >10 years) *

- ◯ <5 years; I understand that implant treatment might not last forever.
- ◯ 5-10 years; I expect that implants can last for a while but not forever.
- ◯ >10 years; implant treatment is expected to last for a long period of time.

13. For the final impression process, do you have a preference for a digital scan, a conventional scan, or depending on the provider's preference? *

- ◯ I'll prefer a state-of-the-art digital impression.
- ◯ I'll prefer a conventional alginate impression
- ◯ I don't have a preference.

14. What concerns or questions do you have about potential complications related to your full-arch prosthesis treatment? *

Please choose three of the following as your highest priority.

Please select 3 options.

- ☐ Pain/swelling from the surgical procedure(s)
- ☐ Transitional stage without the final denture
- ☐ Need for taking many medications for post-surgery care
- ☐ Need for more frequent check-up appointments in the future
- ☐ Increased difficulty of cleaning
- ☐ Increased difficulty of speaking
- ☐ Risk of implant failure (such as implant inflammation)
- ☐ Risk of prosthesis failure (such as denture fracture)
- ☐ Possible change of the bite/ joint relation
- ☐ Other

15. How well-informed do you feel your doctor(s) communicated with you about what to expect and the longevity of your implants? *

- ○ I feel very well-informed; my doctor(s) informed me that the implants and the prosthesis may need to be replaced at some point.
- ○ I feel somewhat informed; my doctor(s) mentioned some information related to the implant/prosthesis longevity, but no details were provided.
- ○ I feel not well-informed; my doctor(s) did not provide information related to the implant/prosthesis longevity throughout the treatment process.

16. **Overall goals and structure of future studies.** The following questions ask about the overall goals and structure of your ideal study. *

*On a scale of 1 to 7 (where 1 is "strongly disagree" and 7 is "strongly agree"), [**Make sure to slide the table all the way to the right to see all score levels, including "7, strongly agree."],** please indicate how much you agree with the following statements:*

	1 (strongly disagree)	2	3	4 (neutral)	5	6	7 (strongly agree)
The study should aim to find out which type of implant-supported prosthesis (denture) works best	○	○	○	○	○	○	○
The study should compare participants receiving the implant-supported prosthesis with those receiving a conventional complete denture (no implants)	○	○	○	○	○	○	○
The study should also test new apps and devices (e.g. robotics) to see if they can improve the way efficacy and success of implant-supported prosthesis is measured	○	○	○	○	○	○	○

17. **Measuring the effect of the new treatment - Part 1.** The following statements suggest ways to measure the results of implant-supported prosthesis (dentures). Please indicate whether you agree that these should be used to show the overall success of the study. *

*On a scale of 1 to 7 (where 1 is "strongly disagree" and 7 is "strongly agree") [**Make sure to slide the table all the way to the right to see all score levels, including "7, strongly agree."**], it is important that the overall success of the study is shown by:*

	1 (strongly disagree)	2	3	4 (neutral)	5	6	7 (strongly agree)
an improvement in quality of life	○	○	○	○	○	○	○
an improvement on activities of daily living and function (chewing, speaking, retention)	○	○	○	○	○	○	○
an improvement on aesthetics from patient's perspective	○	○	○	○	○	○	○
an improvement on aesthetics from patient's perspective	○	○	○	○	○	○	○
a reduction of complications	○	○	○	○	○	○	○

18. **Measuring the effect of the new treatment - Part 2.** The following statements suggest ways to measure the results of implant-supported prosthesis (dentures). Please indicate whether you agree that these should be used to show the overall success of the study. *

*On a scale of 1 to 7 (where 1 is "strongly disagree" and 7 is "strongly agree") [**Make sure to slide the table all the way to the right to see all score levels, including "7, strongly agree."**], please indicate how much you agree with the following statements:*

	1 (strongly disagree)	2	3	4 (neutral)	5	6	7 (strongly agree)
It is important that the study measures the difficulty of the procedure from clinician's perspective to help other clinicians who want to use the same procedure	○	○	○	○	○	○	○
It is important that the study measures cost-effectiveness of the treatment	○	○	○	○	○	○	○

19. Are there any important results, outcomes, or aspects that you think should be measured in patients receiving an implant-supported prosthesis (implant-retained denture) that were not mentioned in the previous questions? *

This content is neither created nor endorsed by Microsoft. The data you submit will be sent to the form owner.

Microsoft Forms
