# Supplementary material for: 1st Global Consensus for Clinical Guidelines for the Rehabilitation of the Edentulous Maxilla: Patient and Cross‐Disciplinary Expert Single‐Round Surveys
Source: Clin Oral Implants Res. 2026 Feb 24;37(Suppl 30):S188–203. doi: 10.1111/clr.70023 (PMC12930135; doi:10.1111/clr.70023)

**SUPPLEMENTARY FIGURES**

For each 7-point Likert scale question, a graph was created reporting medians and interquartile ranges (IQR).

**Suppl. Figure 1. Overall goals and structure of future studies (professional investigations) – from the patient survey. The following questions ask about the overall goals and structure of your ideal study (to improve patient care, information and treatment results).**

**
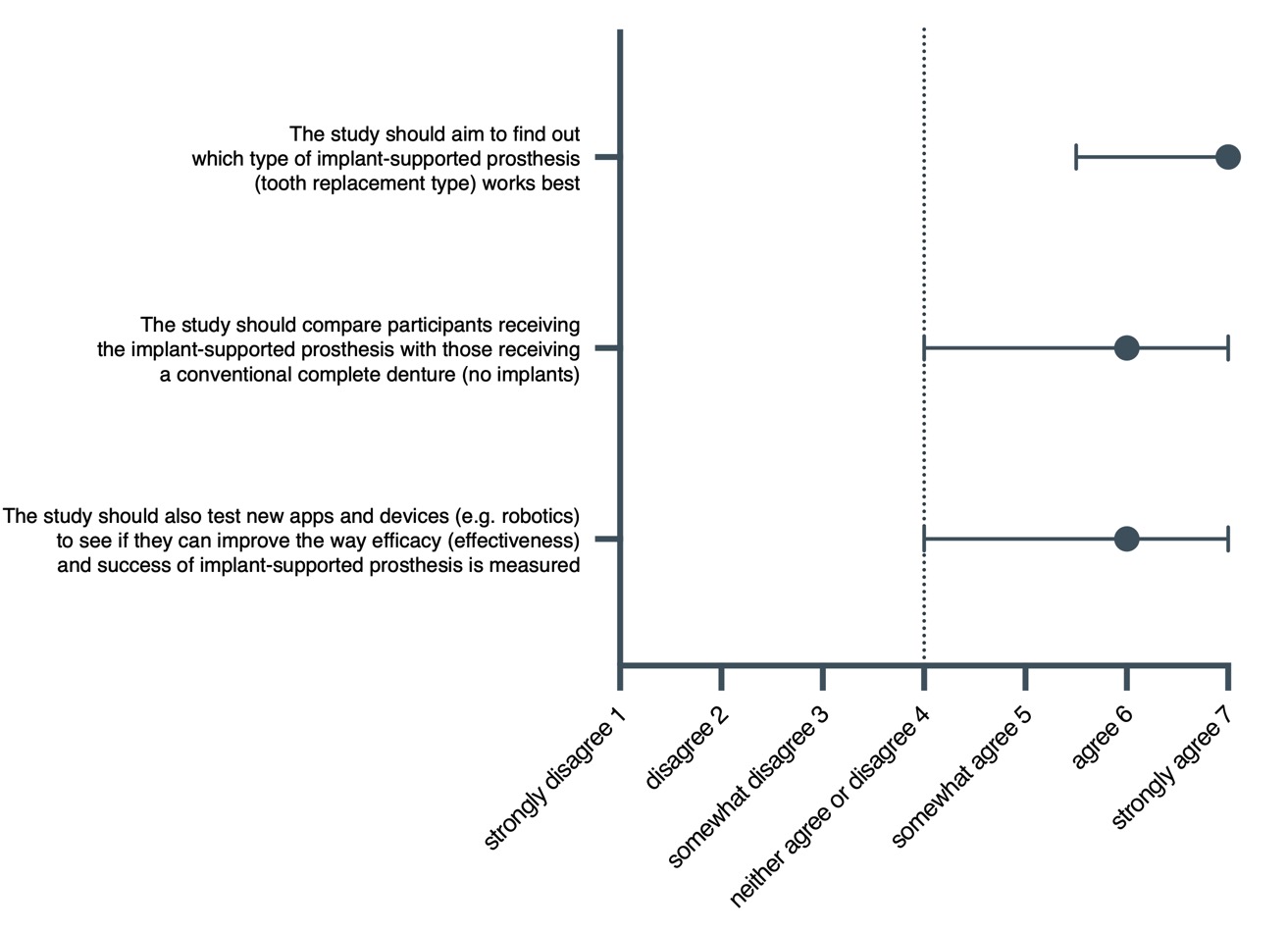
**

**Suppl. Figure 2. Overall goals and structure of future studies – from the cross-disciplinary expert survey. The following questions ask about the overall goals and structure of your ideal study.**


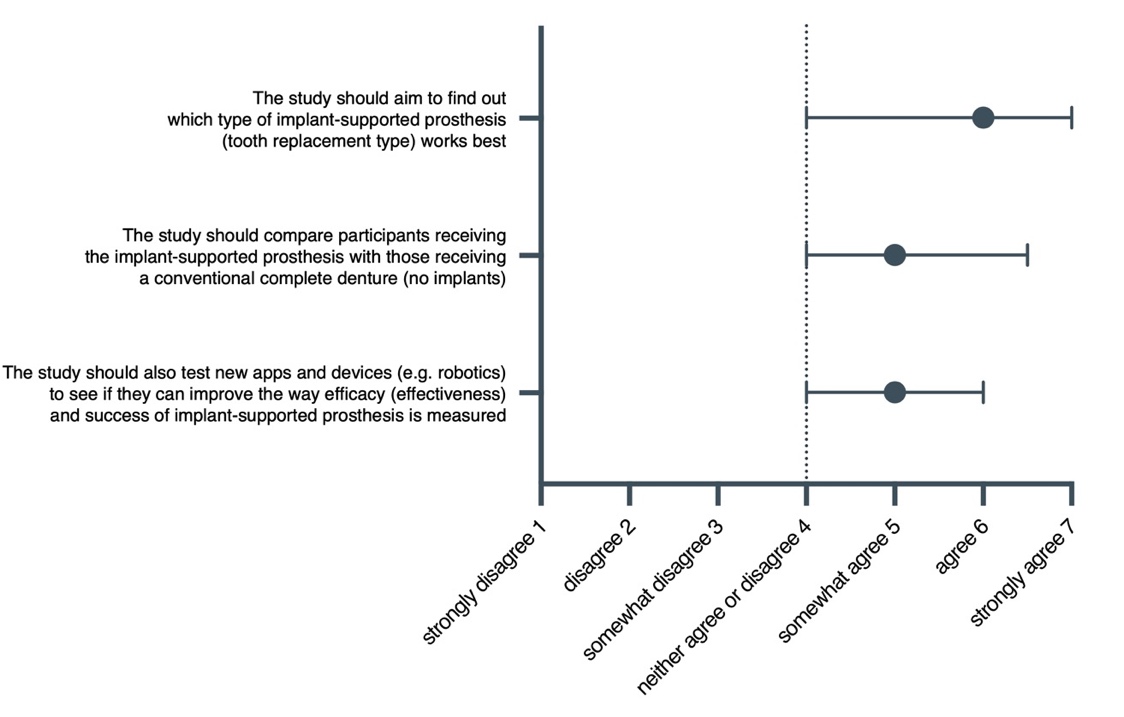


**Suppl. Figure 3. Measuring the effect of the new treatment - Part 1 – from the patient survey. The following statements suggest ways to measure the results of implant-supported prosthesis (tooth replacements). Please indicate whether you agree that these should be used to show the overall success of the study.**

**
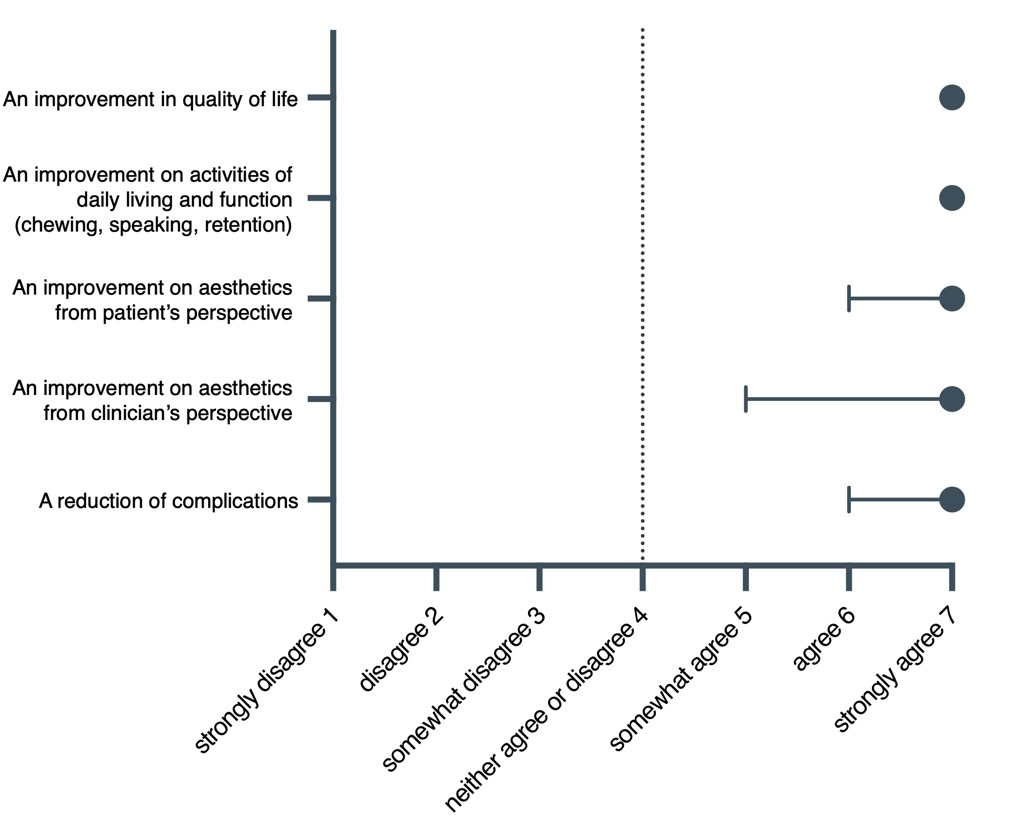
**

**Suppl. Figure 4. Measuring the effect of the new treatment - Part 1 – from the cross-disciplinary expert survey. The following statements suggest ways to measure the results of implant-supported prosthesis (dentures). Please indicate whether you agree that these should be used to show the overall success of the study.**


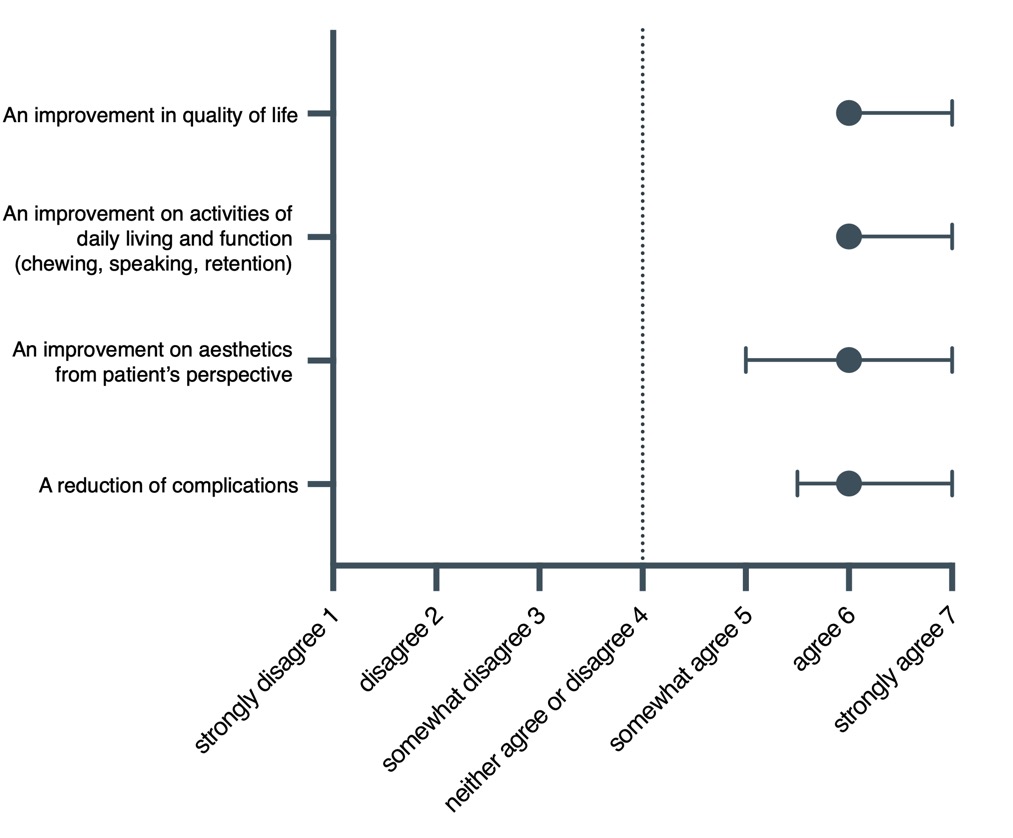


**Suppl. Figure 5. Measuring the effect of the new treatment - Part 2 – from the patient survey. The following statements suggest ways to measure the results of implant-supported prosthesis (dentures). Please indicate whether you agree that these should be used to show the overall success of the study.**

**
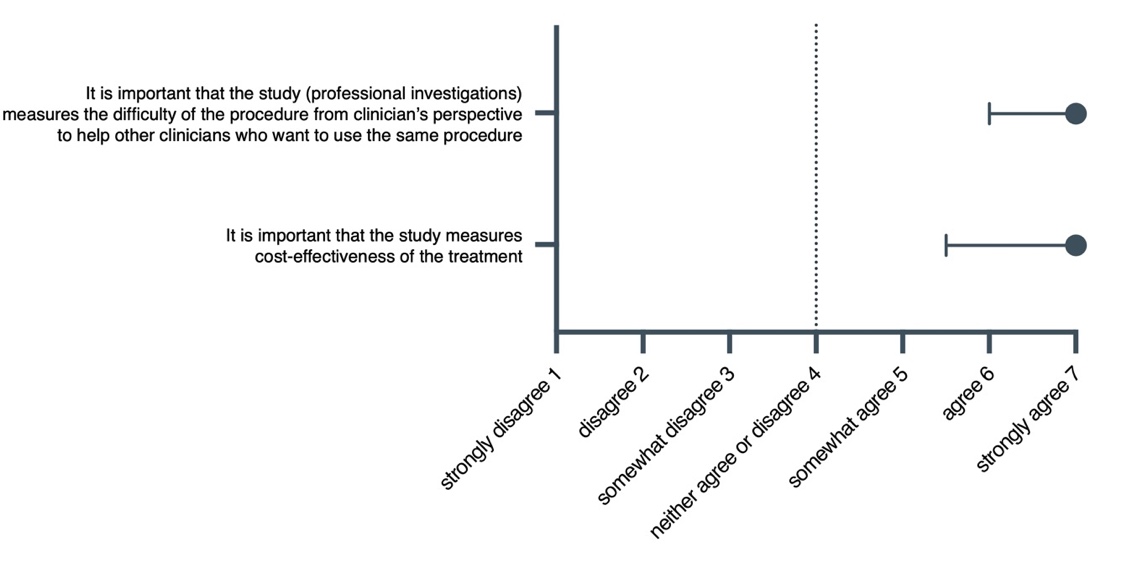
**

**Suppl. Figure 6. Measuring the effect of the new treatment - Part 2 – from the cross-disciplinary expert survey. The following statements suggest ways to measure the results of implant-supported prosthesis (dentures). Please indicate whether you agree that these should be used to show the overall success of the study.**


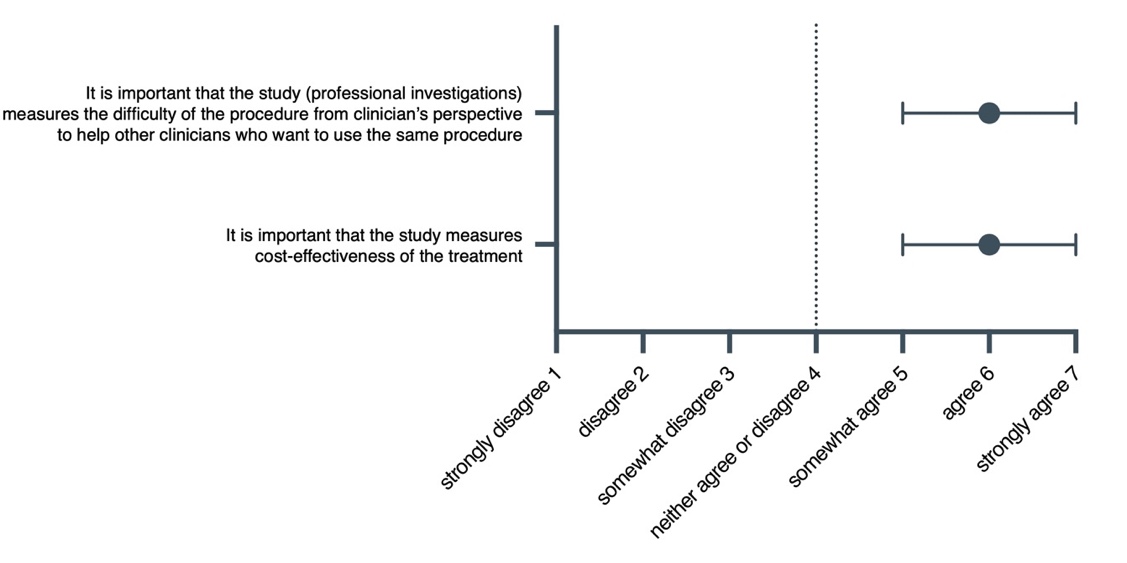

Supplement: Supplementary file 3 — Data S1: clr70023‐sup‐0003‐Figures.docx. [file CLR-37-S188-s002.docx]
